# Dog-ownership and paediatric neurodevelopmental disorders; ‘pawsitive’ impact: a systematic review

**DOI:** 10.1038/s41390-025-04206-7

**Published:** 2025-07-30

**Authors:** Tiarnán Ó Conaill, Ailbhe Whitty, Simon K. Hollingsworth, Anna McGee, Nazia Rafiq, Aaron Coleman, Anna Daly, Aaron Earley, Andre Loo, Aisyah Nizam, James Trayer, Philip Stewart, Aoife Branagan, Eoghan Lally, Edna Roche, Judith Meehan, Eleanor J Molloy

**Affiliations:** 1 Discipline of Paediatrics, Trinity College Dublin, the University of Dublin, Dublin, Ireland; 2 Madra Man, Dog training Dublin, Dublin, Ireland; 3 Trinity Research in Childhood Centre (TRiCC), Dublin, Ireland; 4 Endocrinology Children’s Health Ireland (CHI), Dublin, Ireland; 5 Trinity Translational Medicine Institute (TTMI), St James Hospital, Dublin, Ireland; 6 Neurodisability Children’s Health Ireland (CHI), Dublin, Ireland; 7 Neonatology Children’s Health Ireland (CHI), Dublin, Ireland; 8 Department of Paediatrics, Coombe Women’s and Infant’s University Hospital, Dublin, Ireland

## Abstract

**Background:**

Owning a dog has been associated with improved well-being and this study focused on dog ownership in children with neurodevelopmental disorders (NDD), especially in autism spectrum disorder (ASD).

**Methods:**

This systematic review utilised Preferred Reporting Items for Systematic Review and Meta-Analysis Protocols (PRISMA-P) and three databases, EMBASE, MEDLINE and Cochrane Library, to assess dog ownership and neurodevelopmental outcomes. Paper screening and data extraction were performed in duplicate using Covidence. The five domains of neurodevelopment that were reviewed included cognitive, social and emotional, speech and language, fine motor and gross motor developmental outcomes.

**Results:**

There were 451 papers reviewed and 16 were included in the final analysis. Despite heterogeneous reporting methods, the impact of dog ownership on children with ASD was positive across multiple domains of neurodevelopment. Fourteen studies reported improved emotional regulation and social engagement in children with ASD with a dog. Improvements in cognitive, speech and language function were reported in seven studies. Additionally, in six of the studies, a pet dog improved family dynamics and reduced anxiety levels in parents of children with ASD. The most common study design included in the systematic review was cross-sectional studies, labrador-retrievers were the most commonly reported dog breed. Eight studies reported the presence of an additional household pet.

**Conclusion:**

Dog ownership was a feasible non-pharmacological intervention, as part of a global, multi-disciplinary approach for children with NDD. Large prospective cohort studies could investigate the mechanism by which dogs provide positive changes in the life of a child with ASD and long-term outcomes.

**Impact:**

- This study highlights that dog ownership in children with neurodevelopment disorders is associated with longstanding benefits in neurodevelopmental outcomes and has wider-reaching effects on the child’s family.
- This is the first systematic review examining the effect of dog ownership in this cohort and hopes to progress the field of dog ownership in paediatric neurodevelopmental disorders.
- The lasting impact dogs have on the lives of children with neurodevelopmental disorders should be viewed as a non-pharmacological adjunct to the holistic care of this patient cohort and highlights the potential for implementation of animal-assisted interventions in future treatment plans.

## Introduction

The relationship between humans and dogs has evolved over millennia, with pet ownership deeply embed in modern society. Extensive research has highlighted the potential health benefits of dog ownership during childhood, particularly in reducing the risk of developing chronic conditions such as asthma, food allergies, and obesity in later life.^1–4^ Beyond these well-documented physiological effects, early-life exposure to dogs may also exert wide-reaching influences on neurodevelopment, shaping cognitive, social and emotional outcomes. However, the evidence in this domain remains limited, warranting further investigation into the broader developmental impact of childhood dog ownership.

Autism spectrum disorder (ASD), a neurodevelopmental disorder (NDD), encompasses a range of early-emerging social communication deficits and repetitive sensory-motor behaviours.^5^ It has been estimated that 1 in 100 children worldwide are diagnosed with ASD,^6^ and due to adjustments in diagnostic criteria and other factors, these prevalence estimates have increased over time. ASD is a lifelong developmental disability with a multi-dimensional impact on functioning in childhood. Given the rising prevalence of ASD, investigating the potential connection between neurodevelopmental outcomes and earlier life experiences such as dog ownership could provide a novel intervention in the management of NDD.

Neurodevelopment is a multifactorial process. External stimuli such as owning a dog in early childhood can influence cognitive, social, speech and language outcomes. Extensive research has delved into the positive effects of having an assistance or therapy dog in NDDs.^7–9^ However, there remains uncertainty regarding the influence of continuous pet ownership within a household compared to interactions with trained service or therapy dogs on children’s neurodevelopment. Several studies have attempted to explore this connection; however, the evidence remains inconclusive and fragmented.

This systematic review synthesises the existing literature and provides a thorough understanding of the implications of dog ownership in childhood for those with NDD.

## Methods

### Search strategy

We conducted a comprehensive, systematic search in accordance with the PRISMA guidelines and was registered in PROSPERO (CRD42024621511. Available from https://www.crd.york.ac.uk/PROSPERO/view/CRD42024621511). EMBASE, MEDLINE and Cochrane Library electronic databases were searched up until 11/04/2024. An additional study was included on the prospective examination of existing literature, as it was deemed a seminal paper. Two authors collaboratively generated the search strategy to ensure accuracy and comprehensiveness. The PICO question was ‘Do children with NDDs (patient) have improved outcomes (comparison and outcomes) from dog ownership (intervention)’. Search terms were applied using Boolean strings agreed upon by author consensus (Appendix 1).

### Eligibility/exclusion criteria

Inclusion criteria were: (i) participants with documented dog ownership; (ii) participants with NDD; (iii) dog ownership between foetal life and 18 years of age; (iv) papers with sufficient information regarding neurodevelopmental outcomes (clearly identifying cognitive, social and emotional, speech and language, fine motor or gross motor neurodevelopmental outcomes) (v) papers published between 2009 and 2024 inclusive. Exclusion criteria were (i) articles unavailable as full-text; (ii) articles unavailable in English; (iii) studies that included participants over the age of 18 years of age; (iv) studies involving foetal exposure without subsequent ex-utero exposure; (v) studies involving dog-assisted interventions; (vi) studies which did not differentiate between types of animal ownership; (vii) case reports, conference proceedings, expert opinions, and letters to the editor.

For the purpose of this review, dog ownership was defined as having a dog within the home, with daily exposure of at least 1 h (if specified), and for a period of 4 weeks or longer. This definition included service dogs living within the home as primary exposure in the home was similar to that of a pet dog, but excluded designated therapy dogs as exposure was irregular and provided a novel stimulus. Pet ownership was defined as the keeping of dogs primarily for companionship within a household, while trained dog ownership was determined to be the possession of dogs for specific tasks such as assistance, therapy or service roles. NDD included Apraxia, ASD, Attention Deficit Hyperactivity Disorder (ADHD), Cerebral Palsy, Conduct disorder, Dyslexia, Intellectual Disability, Learning Disorder, and Pervasive developmental disorder not otherwise specified.

### Screening

**Fig. 1:**
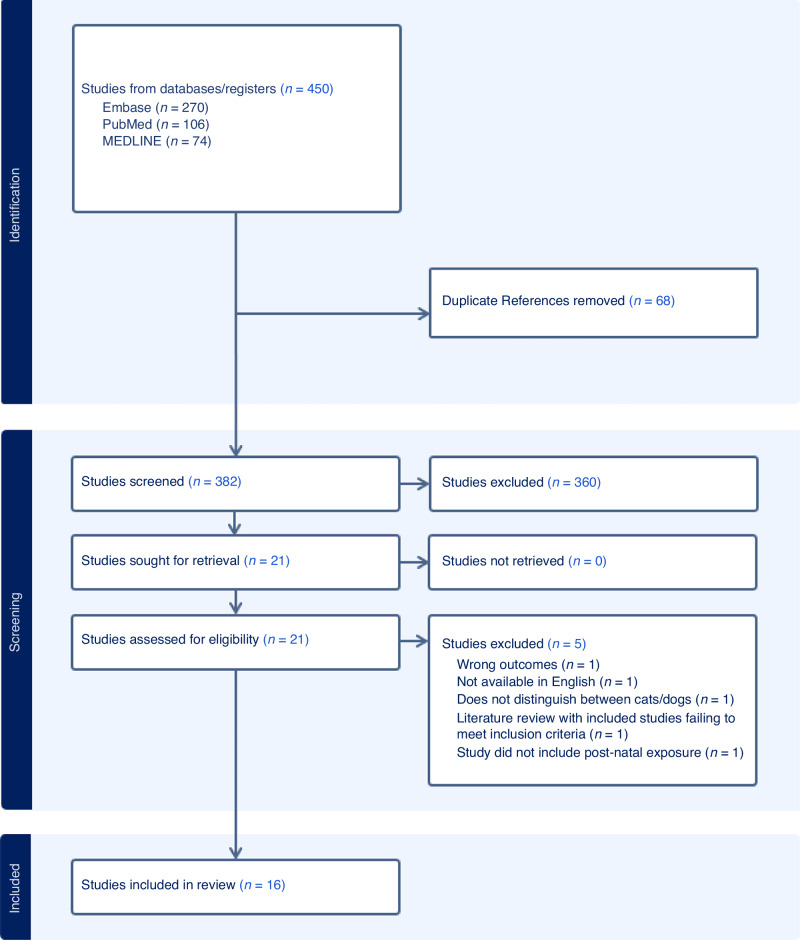
PRISMA flow chart for dog ownership and neurodevelopmental outcome. PRISMA flow-chart for dog ownership and neurodevelopmental outcomes in children with neurodevelopmental disorders. Created by Covidence.

A virtual systematic review management system, Covidence, was used for the screening process. Two independent reviewers screened titles and abstracts. Articles that did not meet the predefined inclusion criteria were removed, and any discrepancies were resolved by a third independent member of the team. The full-text review was conducted in the same manner (Fig. 1).

### Data extraction

Data pertaining to primary, secondary, and tertiary outcomes were extracted by independent members of the team. Primary outcomes included population characteristics such as age, gender and NDD diagnosis. Sex- and gender-based analyses were not conducted due to a lack of reporting in the included studies. Information regarding developmental outcomes was also extracted. These outcomes were distinguished based on the five main domains of development: (i) cognitive; (ii) social and emotional; (iii) speech and language; (iv) fine motor; and (v) gross motor. Secondary outcomes measured included the effects of dog ownership on parents, and scales used to measure the impact of ownership. Tertiary outcomes measured were study design, dog breed, number of dogs in the home and presence of any other animal species within the home. Following the extraction of data, results were compiled into a table to allow trends in outcome effects to be extrapolated.

### Risk of bias

Risk of Bias was assessed using the ROBINS-1 (Risk of Bias in Non-randomised Studies of Interventions) tool. This tool was employed by two independent authors, with a third author available to resolve any conflicts.

## Results

### Screening and outcome reporting

In total, 451 papers were identified from the search strategy outlined above. 68 of these papers were duplicates and therefore removed prior to abstract screening. 383 papers qualified for screening, of which 361 were excluded during abstract/title screening as they were deemed irrelevant. One further study was removed as it was not available in English. Another study was removed from analysis as the contents of the literature review included studies already screened for and did not meet eligibility criteria. This resulted in 16 papers suitable for our review after full-text review (Table 1).

**Table 1:** Demographics of populations included in the systematic review.

Reference	Study design	Sample size	Neurodevelopmental disorders included	Control used	Age (in years)	Gender
(Carlisle^15^)	Cross-sectional descriptive survey	70 (70)	ASD, PDD NOS	-	8–18 (30–59)	65 boys, 5 girls (61 females, 9 males)
(Viau et al.^46^)	Exploratory study (qualitative research)	42	ASD, PDD NOS	-	3–14	37 boys, 5 girls
(Agnew et al.^17^)	Cohort study-semi structured interviews	7 (6)	ASD (4 had secondary ID, 3 had secondary ADHD)	-	4–11 (25–50)	6 boys, 1 girl (6 female)
(Morgan and O’Byrne^11^)	Cohort study-semi structured interviews	9 (9)	ASD	-	Not specified	Not specified
(Grandgeorge et al.^21^)	Observational cohort study	40	ASD (all participants had a learning disability)	Yes	6–34	26 boys, 14 girls
(Carlisle et al.^16^)	Cross-sectional study	764 (764)	ASD, PDD, PDD NOS	Yes	3–18	598 boys (689 female)
(Fecteau et al.^23^)	Case-control study	98	ASD, PDD NOS	Yes	5–10	Not specified
(Tseng^13^)	Cohort study	12 (11)	ASD (11^a^), ADHD (4^a^), ID (7^a^)	-	6–12	10 boys, 2 girls (12 females)
(Rodriguez et al.^18^)	Cross-sectional study	75	ASD, PDD, PDD NOS	Yes	5–17	54 boys, 21 girls
(Wright et al.^22^)	Case-control study	62 (62)	ASD	Yes	2–16	38 boys, 24 girls (5 males, 57 females)
(Burgoyne et al.^24^)	Cross-sectional study	80 (134)	ASD	Yes	0–9	70 boys, 10 girls
(Ward et al.^14^)	Cross-sectional study	73 (152)	ASD (No-to-mild cognitive impairment)	-	12–17	Not specified
(Leighton et al.^19^)	Cross-sectional study	50	ASD	Yes	5–17	(5 male, 45 females)
(Adkins et al.^10^)	Cohort study	10 (10)	ASD	-	8–17	8 males, 2 females
(Hall et al.^12^)	Case-control cross-sectional study	70 (70)	ASD	Yes	3–16	52 boys, 18 girls (14 male, 56 females)
(Harwood et al.^20^)	Multiple Case Study	13(11)	ASD	-	5–12	7 boys, 6 girls (11 females)

Statistics included in brackets indicate figures of parents or caregivers extracted from included studies unless otherwise specified.

*ASD* Autism Spectrum Disorder, *PDD NOS* Pervasive Developmental Disorder Not Otherwise specified, *ADHD* Attention Deficit Hyperactivity Disorder, *LD* learning disability, *ID* intellectual disability.

^a^Figures included depict the number of children with a given neurodevelopmental disorder included in the study.

Developmental outcomes of children exposed to dog ownership were heterogeneous, both in the methods used to measure these outcomes and in the effect of dog ownership on the populations. As a result, the collection of data from studies was similarly disparate, thus hindering statistical analysis of study results and direct comparisons of effects reported between studies.

#### Primary outcomes

The most common neurodevelopmental outcome reported in the studies was social and emotional development of children with NDD, identified in 13 of our 16 studies (Table 2). Cognitive outcomes were reported in six studies, while gross motor and fine motor development were reported in four of the papers (Appendix 2). Speech and language development was the least reported outcome, evidenced in two of the papers.^10,11^

**Table 2:** Improvements in social and emotional developmental outcomes in children with NDD.

Reference	Social and emotional development subcategory			
	Social interaction	Bond and companionship	Problematic behaviour	Mood, stress, calmness, and sleep
(Carlisle^15^)	Increased social skill in assertion	Formed bond to dog. Parent could identify which dog child formed the strongest bond with, if more than one present	-	-
(Viau et al.^46^)	-	-	Decreased problematic behaviours	-
(Agnew et al.^17^)	Increased Community engagement and participation. Effect described as an isolation to freedom	-	Decreased incidence of ‘meltdowns’, aggression, and challenging behaviour	Sensory intervention from dog ownership assisted in managing emotional states Decreased anxiety and panic attacks. Easier to go to sleep, return to sleep and assist in sleepwalking behaviour
(Morgan and O’Byrne^11^)	Increased positive social behaviours and verbal communication. Increased social skills in eye contact, smiling and improved sibling bond. Improved safety in public due to having an assistance dog	Companionship established with dog, ‘cuddled’ or ‘wrapped around’ dog	Decreased problematic behaviours	-

(Grandgeorge et al.^21^)	Increased social skills in ‘offering to share’ and ‘offering comfort’	Some children had ‘privileged relationship’ with the dog	-	-
(Carlisle et al.^16^)	Increased social interaction	Formed an attachment to the dog. Established companionship and affection towards dog	-	Increased relaxation and happiness
(Tseng^13^)	-	-	Decreased aggression	Decreased stress
(Rodriguez et al.^18^)	-	-	Decreased aggression	Decreased stress
(Ward et al.^14^)	-	Established companionship with the dog	-	Decreased depressive symptoms. Decreased episodes of being overwhelmed
(Leighton et al.^19^)	Increased interactions, decreased stigma, and judgement. Increased patient and tolerance in the child	-	-	-
(Adkins et al.^10^)		Formed bond with dog. Caring for the dog became important and part of a routine	-	Increased happiness and pride in taking care of dog
(Harwood et al.^20^)	Increased participation with the world	Established companionship with dog that eased loneliness	-	Dog provided comfort and calming influence. Dog provided a direct sensory intervention and distraction from stress

### Social and emotional development

The most consistently reported effect in this category among the 14 papers was a link between reducing stress/promoting calmness, improving behaviour, improving social interaction with others, and improving mood (Table 2). A reduction in the prevalence of an individual child’s symptoms was seen in three papers.^12–14^ A strong bond between the child and the dog was reported in two papers.^10,15^ The dog improving the child’s companionship with others was reported in two papers.^11,16^ A closer relationship with the dog itself was reported to occur when the dog was introduced at a younger age in one paper.^15^ Finally, improved sleep when paired with a dog was seen in two papers.^17,18^

### Cognitive development

Two papers reported an increase in the child’s independence.^11,17^ Two papers reported an increase in the child’s attention.^11,13^ One paper described an improvement in the child’s compliance with routines, due to the dog’s behaviour when they failed to complete a task.^17^ The same paper also described an increase in engagement with therapy sessions. One paper reported an increased focus on completion and engagement in academic work.^11^

### Speech and language development

A quantitative increase in verbal communication was reported in two papers.^10,11^ This was measured as an increase of child’s expressiveness, either in talking more to others or to the dog. In both of the papers, language development was suggested from the increase in the quantity of words in the child’s vocabulary.

### Gross motor skill development

The two key gross motor developments, observed across four studies were: increased physical fitness and ability to care for the dog (Table 3). In two of the four aforementioned papers, one reported that the child’s exercise level had increased,^16^ while the other highlighted improvement in walking tempo and gait.^19^ The remaining two studies described how the children developed motor skills to care for their dogs, which included feeding and preparing water.^10,20^

**Table 3:** Benefits of dog ownership on family dynamics in families of children with neurodevelopmental disorders.

Reference	Benefits of dog ownership on family dynamics			
	Family benefits	Parental wellbeing	Family outings	Salivary cortisol
(Agnew et al.^17^)	Increased family engagement	Reduced stress during family outings	Increased family outings	-
(Morgan and O’Byrne^11^)	Family were all included	Increased family outings	Increased family outings	-
(Grandgeorge et al.^21^)	More interactions within the family and more cohesion	-	-	-
(Carlisle et al.^16^)	-	Increased relaxation	-	-
(Tseng^13^)	-	Reduced parental stress	-	Reduced cortisol level
(Wright et al.^22^)	-	Reduced stress in intervention group	-	-
(Burgoyne et al.^24^)	Improved relationship and comforting to the family. Provided a focus for fun and play			
(Leighton et al.^19^)	Improved family interactions and wellbeing. Improved overall mood of the family as a source of joy, laughter, and play			
(Adkins et al.^10^)	Improved family life and interactions	-	-	-

(Hall et al.^12^)	-	Parenting stress reduction in: total stress, parental distress, parent-child dysfunctional interactions, and difficult child	-	-
(Harwood et al.^20^)	-	stress free and positive family environment	-	-
(Fecteau et al.^23^)	-	-	-	Reduced morning cortisol

### Fine motor skill development

Two studies reported the improvement of fine motor skills.^19,21^ In both studies, it was noted that there was increased play with the dog (Table 3). Playing with pets is a complex behaviour, which involves fine motor skills, such as manipulating toys and objects. The positive impact in this domain is further substantiated by the involvement of fine motor in a range of other activities, such as caring for the dog.

#### Secondary outcomes

##### Scales used to measure effects

A variable and heterogenous group of both standardised and accredited rating scales, along with purpose written questionnaires were used across all included papers. The most widely used measurement scale was the short form of the Parenting Stress Index (PSI), which was used in four papers.^12,13,22,23^ The Companion Animal Bonding Scale, Lexington attachment to pet’s scale, Autism Diagnostic Interview—Revised and Cortisol Awakening Response, were each used in two papers (Appendix 3). Semi-structured interviews were carried out in two of the papers, and therefore did not objectively measure the effect using a questionnaire or scale.^10,20^

### Effects on parents

This study also examined the effects of dog ownership on family dynamics in families of children with NDD. Improved parental wellbeing was the most reported benefit (Table 4). Additionally, family benefits were reported in six of the studies, which included increased familial engagement, interactions, and improved independence.^10,11,17,19,21,24^ Two papers reported an increase in the number of family outings, which had not previously been possible prior to dog ownership.^11,17^ A reduction in salivary cortisol levels was reported in two papers.^13,23^ Salivary cortisol is a frequently used biomarker of psychological stress.^25^ Conversely, three papers identified challenges in the cost of keeping their dog, alongside two papers quoting parental difficulties in cleaning (Table 5). Other disadvantages to dog ownership on familial dynamics reported included an increased parental responsibility in two papers, and difficulties adjusting to the dog.^20,24^

**Table 4:** Challenges and burdens of dog ownership faced by carers and families of children with neurodevelopmental disorders.

Reference	Challenge of dog ownership faced by carers and families			
	Increased responsibility	Cost and cleaning	Family perception	Adjustment to dog ownership
(Agnew et al.^17^)	-	Cost of training and grooming dog		Challenge in tethering child to dog
(Burgoyne et al.^24^)	Adoption of new activities such as walking and caring for dog	Worry about cost of cleaning and holiday restrictions	Fear of family disapproval of dog	-
(Harwood et al.^20^)	Worry over inappropriate acts to dog (e.g child threatening the dog). Need to juggle needs of dog and child	Worry about future vet costs should dog become ill and volume of dog shedding	-	-

**Table 5:** Improvements in gross motor and fine motor development in children with neurodevelopmental disorders exposed to dog ownership.

Reference	Gross Motor development		Fine Motor development
	Physical Activity and health	Caring for the dog	Playing with pet
(Carlisle et al.^16^)	Dog ownership helped increase exercise	-	
(Leighton et al.^19^)	Child’s ‘irregular gait and walking tempo has improved tremendously by walking with [service dog] on harness regularly’	-	One child experienced improvement to sensory sensitivities ‘through touching [the service dog] and [the service dog’s] care’
(Adkins et al.^10^)	-	Children help with **feeding,** **watering,** **walking** and/or **brushing** the dog (only one child stated that they do not take care of the family dog at all)	-
(Grandgeorge et al.^21^)	-	-	Dog ownership involved object manipulation and mastery of action schemas (i.e. sensorimotor play)
(Harwood et al.^20^)	-	Children prepared water and food for the dog, without their mothers having to prompt them all the time	-

#### Tertiary outcomes

Diverse study designs were employed: seven cross-sectional, four cohort, two case control, one observational, one case study, and one exploratory study were included in our systematic review (Table 1). There was a significant degree of heterogeneity in reporting of dog breeds which hinders accurate interpretation of data. The most common dog breed reported was labrador-retrievers which were evidenced in seven studies (Table 6). Additionally, eight studies reported the presence of an additional household pet (Table 6).

**Table 6:** Tertiary outcomes on dog breeds and presence of other animals in the household.

Reference	Dog breeds	Number of dogs per household	Presence of other animals in household
(Carlisle^15^)	Labrador retriever, ‘Medium-large dogs’ (including terriers, sporting and hounds), small mixed breeds, ‘toy breeds’	-	Cats, fish, farm animals, rodents, rabbits, reptiles, arachnid, birds
(Agnew et al.^17^)	Labrador, Labrador cross, other large dog breed	-	Not specified
(Grandgeorge et al.^21^)	-	-	Cat, hamster, rabbit
(Carlisle et al.^16^)	-	-	Bird, cat, fish, rabbit and reptiles
(Tseng^13^)	Labrador retrievers, Golden retrievers, Labrador/Golden retriever cross, Poodle	-	-
(Rodriguez et al.^18^)	Labrador retrievers, Golden retrievers, or Labrador-Golden retriever crosses	-	Other pets not specified
(Wright et al.^22^)	Labrador Retrievers, Golden Retrievers, German Shepherd Dogs, Cavalier King Charles Spaniels, Miniature Schnauzers, Cocker Spaniels, Sussex Spaniel, Jack Russell Terrier, West Highland White Terrier, Border Collie, Bernese Mountain Dog	-	Not specified
(Ward et al.^14^)	-	-	Cats, rodents, fish, reptiles, amphibians, rabbits, birds
(Adkins et al.^10^)	Rottweller, Pit bull mix, Boxer mix, Labrador chow coon mix, Pomeranian, Australian cobberdog, Chihuahua maltese mix, Goldendoodle, Labrador mix, mini poodle	-	-
(Hall et al.^12^)	Cocker spaniels, Cavalier King Charles spaniels, Golden retrievers, miniature schnauzers, Labradors, Jack Russel terrier, West Highland white terrier, fox terrier, Border collie, Bernese mountain dog), cross-breeds	-	-
(Harwood et al.^20^)	Labradoodle, Border Colie, Jack Russel, Small ‘multiple breed’, Schnauzer, Pug Crosses, Staffordshire Terrier, Boxer, Large ‘multiple breed’, Labrador, Shitzu Maltese Cross	-	Guinea pigs, horse, cat

#### Risk of bias assessment

Several studies were assessed as having bias using the ROBINS-II risk of bias assessment tool. Three studies were identified as having a moderate-to-high risk of bias (Table 7). All three studies were deemed as meeting the criteria for confounding bias and selection bias, with two of the studies identified as having a bias from deviation of intervention.^12,13,22^ Only 42 of the 82 participants in Wright et al. completed follow-up as well as recruiting participants from the Pets and Wellbeing Study (PAWS) cohort which could implicate a participant bias towards benefits of dog ownership. No exclusion criteria were included in Hall et al., resulting in a wide variety of family characteristics, as well as recruiting participants from the PAWS cohort. In Tseng et al.'s study, participants completed follow-up both prior to and after a period of COVID-19 restrictions, which may have impacted the perception of affected families on the child’s development.

**Table 7:** Studies identified as moderate-to-high risk of bias.

Reference	Confounding bias	Selection bias	Class of Intervention bias	Bias from deviations of intervention	Incomplete outcome data	Selective measurement of outcomes	Selective outcome reporting	Overall bias
(Tseng^13^)	**✓**	**✓**	✗	✗	**✓**	✗	✗	**✓**
(Wright et al.^22^)	**✓**	**✓**	✗	**✓**	✗	✗	✗	✗
(Hall et al.^12^)	**✓**	**✓**	✗	**✓**	✗	✗	✗	✗

Tseng et al.: Some participants had completed follow-up assessment prior to March 2020, while other participants were first enroled in October 2020 during COVID-19 restrictions. This may have impacted the perception of affected families on their child’s development. Selected participants were also previously on waiting lists to receive a dog, which have increased the likelihood of preconceived beliefs of the benefits of dog ownership. Additionally, assessors were not blind to the intervention.

Wright et al.: Participants were recruited from the PAWS cohort, which could implicate a participant bias towards to benefits of dog ownership. Only 42 of the 82 participants completed follow-up

Hall et al.: No exclusion criteria were included resulting in a wide variety of family characteristics. Participants were recruited from the PAWS cohort, which could implicate a participant bias towards to benefits of dog ownership. Only 52.4% of the intervention group took part in long-term follow-up.

**✓** = High Bias Risk ✗ = Low Bias Risk.

## Discussion

This systematic review evaluated neurodevelopmental outcomes and demographics of dog ownership in paediatric NDD and found largely positive associations, such as improvements in mental health, physical activity and social connections, which points towards a beneficial influence of dog ownership. Although a number of studies lacked control cohorts, the implementation of dog ownership into multi-disciplinary strategies in the management of NDD (cognitive behavioural and occupational therapy) could ameliorate negative behaviours, improve social functioning, enhance skill acquirement, and alleviate parental stressors.

The majority of studies focused on social and emotional outcomes in children with NDD. Berry et al. hypothesised that the easily interpretable behaviours of dogs facilitates engagement of children with NDD, enabled social interaction with dogs in actions that are predictable and repetitive, such as playing fetch or providing commands.^26^ Moreover, the ability of children to respond to these social and emotional cues could aid in the transfer of these skills into more subtle human interactions.^27^ These theories provide a basis for the findings of this systematic review that dog ownership can enhance social interaction and improve social skills in children with NDD.^11,15,16,19,21^

Gross motor, fine motor, cognitive, and speech and language development were explored to a lesser extent than that of social and emotional outcomes in studies included in this systematic review. Two studies identified improvements in speech and language development, which could be due to repetition of words during dog training; Morgan et al. reported that one child’s first word was their dog's name.^11^ However, non-verbal reasoning and skills were predominantly investigated in studies, potentially due to dog behaviour and interaction consisting of non-verbal actions.^28^

Interaction and play with pets is a complex behaviour, consisting of object manipulation and repetition of learned actions.^21^ This is especially significant in ASD as physical interactions can often be avoided due to difficulties in gross motor functioning.^10^ Additionally, low motor performance is related to a higher prevalence of anxiety and low self-esteem.^29^ Improvements in physical activity, such as that documented by Leighton et al.^19^ is also important, as obesity prevention programs in children with NDD are rarely constructed with a family-based approach or tailored to functioning of children with NDD.^10^ Therefore, the potential improvements in gross and fine motor development brought about through dog ownership could have implications not only in terms of neurodevelopment but also in the reduction of comorbid conditions.

Dog ownership can have a profound beneficial impact on family dynamics in families of children with NDD. The positive family environment described by Harwood et al. as well as Adkins et al. reporting of reduced parental stress, the impact of dog ownership on families of children with NDD is wide-reaching.^10,20^ In a similar fashion to the effect of dog ownership on children with NDD, dogs could provide a social support mechanism to parents.^22^ Indeed, Weis et al. recommended a focus on social support interventions to improve carer functioning and coping mechanisms, something which dog ownership has the capacity to provide.^30^ These findings are particularly important as a number of previous research has either failed to identify effects of dog ownership on parental functioning or employed alternative interventions to improve parental stress.^22^

The cost and challenges of dog ownership on families, however, cannot be discounted in light of the overwhelming positives. The addition of service dogs could also contribute to parental and carer workload. However, as highlighted by both Morgan et al. and Burrows et al. the benefits accrued from dog ownership far outweighed challenges faced by families; the alleviation of challenging behaviours.^11,31^ The integration of the care for the dog into routine is essential for the full realisation of the benefits dog ownership provides to families of children with NDD.

During our analysis, it became apparent that the methods of evaluating the effects of dog ownership in children with NDDs are very heterogeneous in nature, involving many different questionnaires as well as interviews. The heterogeneous nature of interviewing and questionnaires is especially apparent in evaluating the effect of dog ownership. The use of standardised measurement scales in literature could enhance effect-outcome comparisons and facilitate meta-analytics on the subject. The PSI was the most documented measurement scale, which has been widely used in previous research as a means for evaluating the effect of childhood intervention on parents.^32^ A wider consensus on effect scales and interviewing style likely would improve the homogeneity of reporting of studies and outcomes to facilitate improved analysis of results.

Although animal-assisted interventions (AAI) were excluded from analysis in this systematic review, numerous studies have identified potential benefits from brief daily interactions with animals as a form of therapy. In particular, Ang et al. found an improved quality of life, reduced mood disturbance and decreased cortisol concentrations in children with NDD exposed to AAI.^33^ The length of time spent interacting with the pet could play a significant role in the development of increasingly more complex social interactions over time. Only three studies reported on the length of time spent with the dog, with Agnew et al. and Ward et al. the only studies to report length of pet ownership (2.5 years and 3.91 years, respectively).^14,17,18^ One interesting concept explored by Grandgeorge et al. is that the arrival of a new stimulus provided by AAI may be potentially more beneficial than constant exposure seen in dog ownership, as children with NDD could prefer novel stimuli as opposed to familiar ones.^21^ This, however, may also depend on the type of constant exposure being referred to, as the age of the dog and level of child bonding with the dog would also play a role in validating whether the novel stimulus is more effective, or if this depends on the initial exposure seen in dog ownership.

Despite ASD being one of many NDDs included in our eligibility criteria, 15 of the 16 included studies feature ASD alone as the disorder of interest. This is understandable, considering the significant burden of ASD worldwide.

However, other NDDs are equally of interest. ADHD has been shown to have a global prevalence of the order of 3.4%, emphasising the need for data on outcomes in those with NDD other than ASD.^34^ All varieties of NDD have far-reaching implications, extending long beyond childhood into adult life and impacting various areas of functioning.^35^

Given the strong focus on ASD in the current literature, it is impossible to make a general recommendation pertaining to dog ownership and its effect on outcomes for those with NDD other than ASD. Certainly, dog ownership has been reported to have positive effects on outcomes for children with ASD, particularly with their social and emotional development in terms of attachment, emotional regulation, and communication. A stress reduction, both subjectively and objectively, has also been reported for the parents of children with ASD who chose to welcome a dog into their home. Even so, care must be taken when recommending dog ownership to these families, and due consideration given to the investment of time, finances and emotion required when introducing a dog to the home.

Benefits of dog ownership extend beyond neurodevelopmental outcomes. In particular, dog ownership has a protective effect against childhood food sensitisation, risk of asthma development and mental well-being.^1,2,36^ Interestingly, dog exposure at specific timepoints in a child’s life can have differing effects on immune development; exposure in infancy can reduce the development of atopic conditions, while meta-analyses have demonstrated positive correlations between dog ownership and non-atopic childhood asthma.^37–39^ One possible explanation could be that early dog-exposure provokes immunological maturation and a skewing of inflammatory signalling towards a type-1 mediated response.^39^ Moreover, these findings could add weight to the hygiene hypothesis, with a lack of exposure to microbial diversity and antigens leading to immunological dysfunction.

The effect of pet ownership on the childhood microbiome has been gaining acclaim in recent literature. Human and pet interactions can provoke changes in their respective microbiome, positively and negatively, with neonatal exposure increasing gut microbial diversity.^40,41^ Pérez-Serrano et al. identified dental plaque microbiota of dog owners could act as a reservoir of antimicrobial resistance genes due to horizontal transfer of microbial species, which could have implications for treatment options in immunocompromised paediatric populations.^42^ Conversely, studies have demonstrated that pet-induced microbiome changes having a protective role against cardiometabolic diseases, promote beneficial microorganisms and early life exposure has been postulated to play a protective factor against the development of Crohn’s Disease.^43,44^

Our study has several strengths; a systematic approach was followed, using the PRISMA reporting guidelines, and a broad search strategy was employed. However, there are some limitations to this systematic review. A significant heterogeneity was identified in included studies, most notably in the reporting of neurodevelopmental outcomes, diagnostic tools employed, study demographics and the use of service dogs. These diversities precluded our ability to perform a meta-analysis and hampered our ability to synthesise results. Due to limited sex- and gender-specific data, we were unable to assess differential effects of dog ownership on developmental outcomes. This limits the generalisability of our findings.

In contrast, several studies only included ASD populations or failed to recruit a diverse cohort. For a more complete understanding of the effects of dog ownership on NDD, a more inclusive search strategy could look to identify case reports and case series on other NDD as well as including the effect of antenatal exposure. Moreover, a number of included studies lacked controls and employed a subjective means of outcome measurement, namely through questionnaires. This, in tandem with the cross-sectional nature of the majority of included studies, implies a significant subjectivity of result reporting and thus limits the generalisability of our results.

The majority of studies included children with ASD as the primary NDD being investigated. Future research should aim to incorporate a wider range of NDDs, which may reveal previously unknown effects of dog ownership in less-studied NDDs. In addition, our review highlights that there is significant heterogeneity between studies in terms of outcome measures, which poses issues when trying to collate data and draw conclusions in systematic reviews such as ours. We suggest that future studies should use standardised outcome measures, thus allowing for data to be accurately compared and improving the generalisability of results. Similarly, many studies included in our review did not contain an appropriate control to measure dog ownership against. Thus, another recommendation of ours is for future studies to include a control arm, such as a robotic dog or a different animal, to act as a comparison. In combination, the incorporation of these suggestions in future studies would allow more definitive recommendations to be made regarding dog ownership as an intervention for families living with NDD.

Another possible avenue of interest could be the identification of molecular pathways between gut-microbiota and inflammatory signatures of children exposed to dogs in early life and its link to neurodevelopmental outcomes. Further studies on dog ownership and the microbiota, such as the work carried out by Gómez-Gallego et al. who investigated the potential for dogs as a probiotic in children, are needed to advance this space further.^45^

In conclusion, we have highlighted that dog ownership in children under the age of 18 is associated with improved neurodevelopmental outcomes, and is also associated with improvement of mood, behaviour, stress, and cognitive functioning. Importantly, we have outlined that dog ownership has far-reaching benefits for both the children and their families and caregivers. It is important to recognise the lasting impact these dogs have on the lives of children with NDDs, and we must endeavour to engage in further research in this area to improve outcomes for such children.

## Supplementary information

Appendix 1

Appendices 2 and 3
