# Supplementary material for: Dog-ownership and paediatric neurodevelopmental disorders; ‘pawsitive’ impact: a systematic review
Source: Pediatr Res. 2025 Jul 30;99(1):77–87. doi: 10.1038/s41390-025-04206-7 (PMC12920082; doi:10.1038/s41390-025-04206-7)
Supplement: Supplementary file 1 — Appendix 1 [file 41390_2025_4206_MOESM1_ESM.pdf]

Appendix

Appendix 1. Search Strategy

Category 1: 'Dog[mh]', 'Canine[mh]', 'Pet NOT radiotracer NOT ligand NOT protein NOT radioligand NOT scan NOT probe NOT tracer NOT imaging[mh]'
Category 2: 'Foetal[mh]', 'Neonate[mh]', 'Newborn[mh]', 'Child[mh]', 'Infant[mh]', 'Adolescent[mh]'
Category 3: 'Autism Spectrum Disorder[mh]', 'Learning disability[mh]', 'Attention deficit disorder[mh]', 'ADHD[mh]', 'Learning Disability[mh]', 'Neurodevelopmental disorder[mh]', 'Cerebral Palsy[mh]', 'Intellectual disability[mh]', 'Conduct disorder[mh]', 'Apraxia[mh]', 'Dyslexia[mh]', 'Pervasive Developmental Disorder[mh]'
Boolean string: Category 1 AND Category 2 AND Category 3
MeSH headings (mh). A replicable search strategy was used for Ovid and Embase databases
